# Morphometric evaluation of the mandibular lingual foramen using cone-beam computed tomography: a retrospective study

**DOI:** 10.1186/s12903-026-09133-5

**Published:** 2026-07-01

**Authors:** Ayse Tas, Tuhfe Celikkol, Yasemin Oztoprak, Nelli Agbulut, Kader Aydin

**Affiliations:** 1 Faculty of Dentistry, Department of Oral and Maxillofacial Radiology, Istanbul Medipol University, Cibali, Atatürk Blv. No:27, Unkapanı/Fatih, Istanbul, 34083 Turkey; 2 Institute of Health Science Department of Oral and Maxillofacial Radiology, Istanbul Medipol University, Istanbul, Turkey; 3 Private Practice, Istanbul, Turkey

**Keywords:** Mandibular lingual foramen, Cone-beam computed tomography, Anatomical variation, Dental implant planning, Anterior mandible, Morphometric analysis

## Abstract

**Background:**

The mandibular lingual foramen (MLF) is a clinically significant structure due to its proximity to neurovascular bundles. Variations in its morphology pose risks of hemorrhage and neurosensory injury during anterior mandibular surgery. The aim of this study was to evaluate the morphometric characteristics of the MLF using cone-beam computed tomography (CBCT) and analyze its relationship with age and gender to define safe surgical boundaries.

**Materials and methods:**

A retrospective analysis was performed on 486 CBCT scans obtained from adult patients. Measurements included the number of lingual foramina, foramen diameter, canal length, distances from the alveolar crest and mandibular base, and total bone height. Gender-based comparisons were performed using Student’s t-test, and correlations with age were assessed using Spearman’s correlation analysis. Inter- and intra-observer reliability were evaluated using intraclass correlation coefficients (ICC).

**Results:**

A single lingual foramen was observed in 62.8% of cases, while 31.9% presented with two foramina, 4.9% with three, and 0.4% with four foramina. Male patients exhibited significantly greater vertical and horizontal bone measurements, including distances from the alveolar crest and mandibular base, total bone height, and canal length (*p* < 0.05). No significant gender difference was observed in foramen diameter. Age showed no correlation with foramen diameter; however, a weak but significant negative correlation was detected between age and canal length (*p* = 0.045). Inter- and intra-observer reliability values were excellent (ICC > 0.98).

**Conclusions:**

The mandibular lingual foramen exhibits considerable anatomical variability influenced by gender and age. Male patients present with larger bone dimensions, while age-related bone resorption leads to a reduction in canal length without affecting foramen diameter. These findings emphasize the importance of individualized CBCT-based evaluation prior to surgical procedures in the anterior mandible to minimize the risk of complications.

## Introduction

The mandibular lingual foramen (MLF) represents an important anatomical structure that must be carefully considered during surgical procedures performed in the anterior mandible. This region contains neurovascular structures that may be at risk during interventions such as dental implant placement, bone graft harvesting, and genioplasty. Inadequate preoperative assessment of this area may result in serious complications, including hemorrhage and neurosensory disturbances [1].

The lingual foramen, commonly located superior or inferior to the genial tubercles, serves as a passage for branches of the sublingual and submental arteries. Previous studies have reported a prevalence exceeding 90%, with considerable variability in the number, size, and course of the lingual canals. These anatomical variations are often not detectable using conventional radiographic techniques, increasing the potential for intraoperative complications [1–3].

Cone beam computed tomography (CBCT) has been widely accepted as a reliable imaging modality for the evaluation of the anterior mandible due to its ability to provide high-resolution three-dimensional images with accurate visualization of osseous structures. CBCT allows precise assessment of the lingual foramen morphology, canal trajectory, and its spatial relationship with adjacent anatomical landmarks such as the alveolar crest and mandibular base [2, 4]. Previous studies have demonstrated that the presence of multiple lingual foramina or wider canals, particularly in the canine region, may increase the risk of bleeding and compromise surgical safety [5].

In addition to anatomical variability, several studies have reported gender-related differences in the morphology of the lingual foramen, with males generally presenting larger canal diameters and more complex anatomical configurations [6–8]. These findings emphasize the importance of individualized radiographic evaluation prior to surgical intervention in the interforaminal region.

Therefore, the aim of the present study was to retrospectively evaluate the morphometric characteristics of the mandibular lingual foramen using CBCT imaging. Specifically, the number of foramina, canal length, entry and exit diameters, and their distances to key anatomical landmarks were analyzed in relation to age and gender. The results of this study aim to contribute to the identification of safe surgical boundaries and to support more predictable planning of surgical procedures in the anterior mandible.

## Materials and methods

### Study design and ethical approval

#### Study design

This study was designed as a retrospective, cross-sectional, and single-center investigation to evaluate the morphometric characteristics of the mandibular lingual foramen (MLF).

#### Study site and duration

The study was conducted at the Department of Oral and Maxillofacial Radiology, Istanbul Medipol University Faculty of Dentistry, Istanbul, Turkey. The research material was retrospectively selected from the institutional imaging archive, encompassing CBCT scans of patients who underwent CBCT imaging between January 2024 and August 2025.

#### Ethical clearance

The study protocol was reviewed and approved by the Non-interventional Clinical Studies Ethics Committee of Istanbul Medipol University (Date:28/08/2025, Decision Number: E-10840098-202.3.02-5829). All procedures were conducted in strict accordance with the ethical principles outlined in the Declaration of Helsinki. Prior to the CBCT analysis, informed consent was obtained from all patients for the use of their radiographic data for research purposes. To ensure participant privacy, all CBCT scans were fully anonymized by assigning a unique identification code to each subject prior to data analysis.

#### Study population and sampling

A power analysis was performed priorly, using OpenEpi (Open-Source Project in Epidemiologic Computing, version 3.01). Based on a medium effect size (Cohen’s d = 0.5), 90% statistical power, and a 5% margin of error for two-tailed comparisons, the minimum required sample size was calculated as 198 subjects.

To ensure a more comprehensive morphometric evaluation, a consecutive sampling strategy was employed. During the 20-month study period, all patients who presented to the hospital were screened; those who met the inclusion and exclusion criteria resulted in a final study population of 486 participants. This final sample size (*n* = 486) significantly exceeds the minimum statistical requirement, thereby enhancing the precision and reliability of the study findings.

### Inclusion and exclusion criteria

CBCT scans of patients older than 18 years old were analyzed. Fully edentulous, partially dentate or completely dentate mandibles were considered if anatomical landmarks were identifiable. Exclusion criteria encompassed presence of bone pathologies (e.g., cysts, tumors), severe periodontitis a history of surgical intervention within the field of this study, impacted teeth or foreign bodies in the interforaminal region, patients with syndromic or congenital conditions affecting the jaws, any history of medications affecting bone metabolism (e.g., bisphosphonates, denosumab), scans with motion artifacts, low resolution, or incomplete visualization of the mandibular interforaminal region or inferior mandibular border.

### Data collection tools and techniques

CBCT scans were acquired using the Planmeca ProMax 3D classic (Planmeca OY, Helsinki, Finland) by a single trained radiologic technician. Patients were positioned using a head-chin stabilizer and instructed to remain motionless. For standardized orientation, the CBCT volumes were aligned while the Frankfort Horizontal Plane was parallel to the floor and the mid-sagittal plane was perpendicular. Acquisition parameters were set following the guidelines and optimized for image quality and radiation safety (320 μm voxel size; 4.8 mA; 99 kVp). Fields of view were sufficient to cover the entire anterior mandible. The images were reviewed using Planmeca Romexis dental software (Version 6.5.1, Planmeca OY, Helsinki, Finland). All scans were assessed on the same LED monitor, positioned approximately 40–50 cm from the observer. For standardized morphometric assessment, multi-planar reconstruction (MPR) was utilized. The axial plane was oriented at the level of the genial spines to define the midline, and cross-sectional (sagittal) slices with a fixed thickness of 1 mm were used for primary measurements. Image assessments were performed in a dimly lit room with optimized tonal adjustments to enhance visibility.

The symphyseal area, defined as the region between the two mental foramina, was the primary focus of all assessments. Data collection utilized both an axial slice, oriented at the level of the genial spines to define the midline, and a corresponding sagittal slice to assess the region of interest, typically using three slices.

The scans were independently evaluated by two Oral and Maxillofacial Radiologists with 8 and 25 years of experience in their field, respectively. Due to the large sample size of the study (*n* = 486), two independent observers were utilized to ensure the efficiency, reliability, and consistency of the morphometric assessments. Prior to data collection, inter- and intra-examiner calibration was performed. To assess intra-examiner and inter-examiner reliability, 10% of the total sample (*n* = 48) was randomly selected and re-measured by both examiners four weeks after the initial assessment. Subsequent scans were assessed individually. In cases where the observers were uncertain, a senior expert researcher, who is a Professor of Oral and Maxillofacial Radiology with 25 years of experience, reviewed the images and provided a final determination. The reliability was statistically confirmed using the Intraclass Correlation Coefficient (ICC) (intra-examiner ICC>0.98; inter-examiner ICC>0.98). To minimize observer fatigue, a maximum of fifteen scans were analyzed per day. Prior to independent evaluation, the researchers underwent calibration by jointly reviewing 20 CBCT images.

**Fig. 1:**
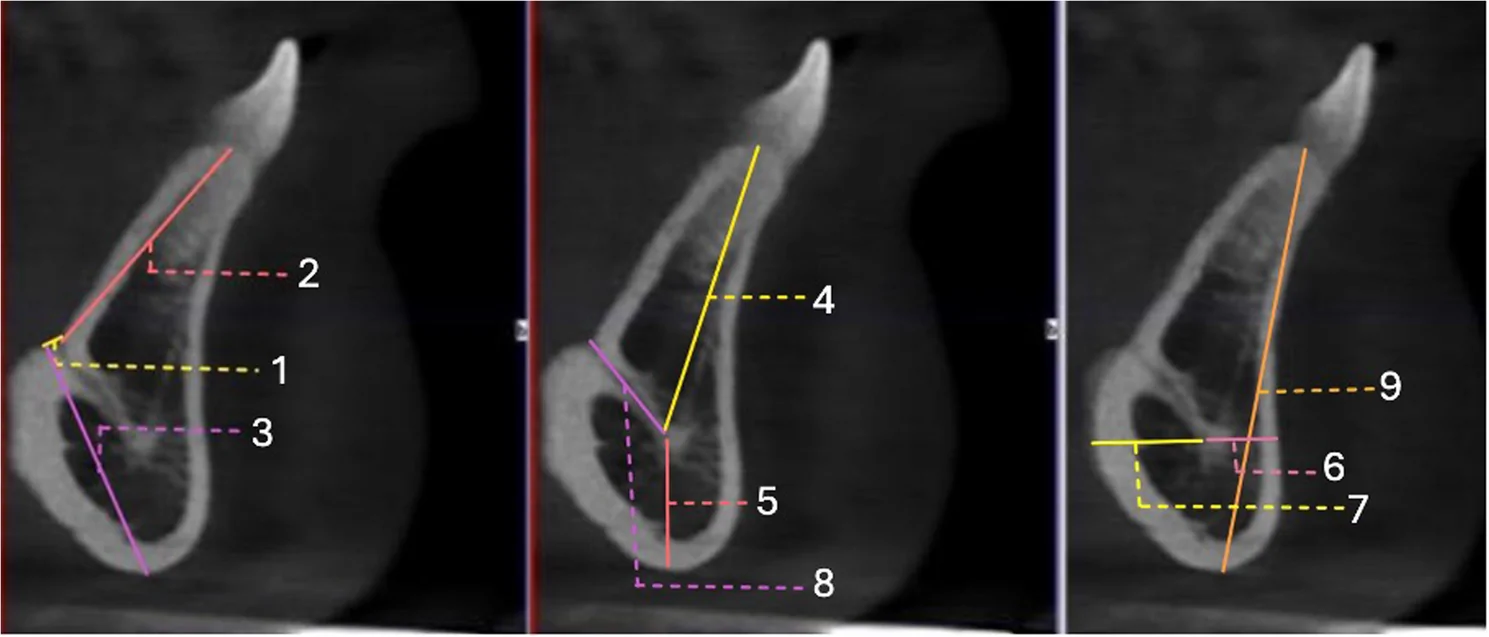
Sagittal CBCT section illustrating anatomical reference lines and morphometric measurements of the mandibular lingual foramen. 1: Diameter of the lingual foramen; 2: Distance from the alveolar crest to the canal’s entrance; 3: Distance from the inferior mandibular basis to the canal’s entrance; 4: Distance from the alveolar crest to the canal’s outlet; 5: Distance from the inferior mandibular basis to the canal’s outlet; 6: Distance from the canal’s outlet to the buccal cortex; 7: Distance from the canal’s outlet to the lingual cortex; 8: Total canal length; 9: Total bone height

Total number of lingual foramina present in the symphyseal area (area between the two mental foramina) was recorded. In patients where multiple foramina or canals were present, the measurements of the most superior foramen were recorded and used for analysis, as this represents the most critical safety margin for surgical procedures. Nine measurements [4] were recorded in millimeters as shown in Fig. 1:

1. Diameter of the lingual foramen
2. Distance from the alveolar crest to the canal’s entrance
3. Distance from the inferior mandibular basis to the canal’s entrance
4. Distance from the alveolar crest to the canal’s outlet
5. Distance from the inferior mandibular basis to the canal’s outlet
6. Distance from the canal’s outlet to buccal cortex
7. Distance from the canal’s outlet to lingual cortex
8. Total canal length
9. Total bone height

**Fig. 2:**
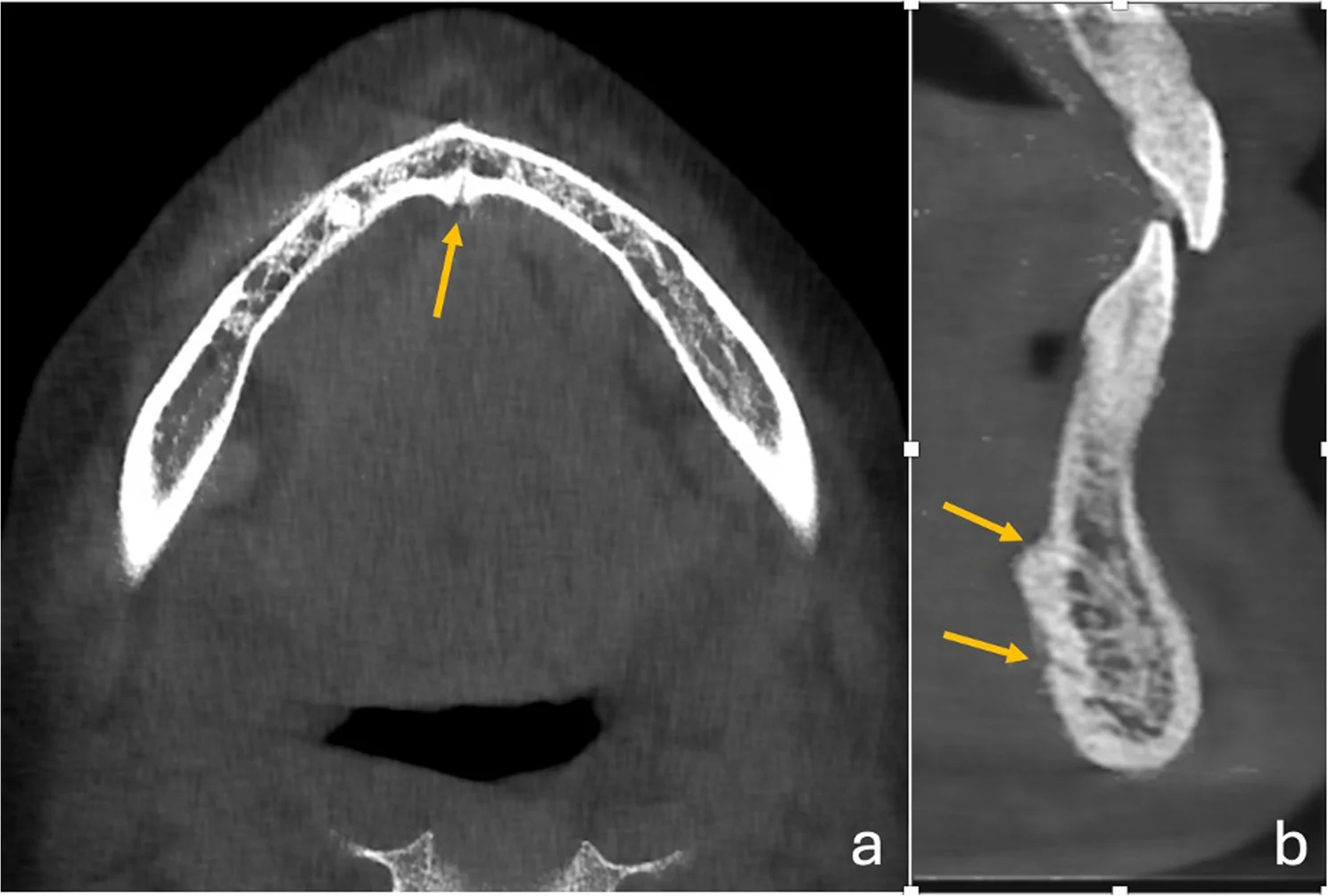
Multi-planar identification and counting of mandibular lingual foramina. **a** Axial CBCT view showing the entry point of the lingual foramen on the mandibular midline. **b** Corresponding cross-sectional (sagittal) view of the same patient, where arrows demonstrate the vertical arrangement of multiple foramina, allowing for an accurate count and independent measurement of each canal

To ensure the accurate identification and counting of multiple foramina, a multi-planar assessment was routinely performed for each subject. As demonstrated in Fig. 2, the axial plane (Fig. 2a) was utilized to confirm the entry point of the foramina on the lingual cortex at the midline. Simultaneously, the cross-sectional (sagittal) plane (Fig. 2b) provided a detailed visualization of the vertical distribution of the canals. This dual-plane approach allowed for the clear distinction between single and multiple foramina (e.g., superior and inferior lingual foramina) and ensured that no accessory canals were overlooked during the morphometric evaluation.

### Statistical analysis

Data were analyzed using statistical software (jamovi version 2.7.6.0). Descriptive statistics were calculated for each study variable, and continuous variables were presented as means and standard deviations. Prior to inferential analyses, the normality of continuous variables was assessed visually using Q-Q plots and histograms and numerically using the Shapiro-Wilk test. The homogeneity of variances was evaluated using Levene’s test. Comparisons between genders were performed using Student’s t-test for variables satisfying the equal variances assumption. For variables in which Levene’s test indicated a significant violation of this assumption (*p* < 0.05), Welch’s t-test was applied instead. Given the large sample size (*n* = 486), Welch’s t-test was preferred over non-parametric alternatives, as the Central Limit Theorem supports the robustness of parametric inference under these conditions. Spearman’s rank correlation coefficient was used to assess the relationships between patient age and the diameter of the lingual foramen, as well as total canal length, given the non-parametric nature of these associations. Statistical significance was set at *p* < 0.05.

## Results

A total of 486 CBCT scans were included for analyses, which comprised scans from 280 female and 206 male patients. Age range is 18–83 years, with a mean of 43.6 ± 16.2 years. Age distribution did not significantly differ among genders (*p* = 0.131).

As the variables under investigation were continuous quantitative measurements, intra-observer reliability was evaluated for each examiner using the Intraclass Correlation Coefficient (ICC). The ICC values demonstrated excellent repeatability, with average overall ICCs of 0.98 and 0.98 for Researcher 1 and Researcher 2, respectively. Inter-observer reliability was also assessed using the ICC (2,1) model, based on a two-way random-effects analysis with absolute agreement. The ICC values for absolute agreement and consistency were 0.98 and 0.98, respectively, indicating excellent inter-observer reliability.

Basic descriptive analyses for all parameters and gender-wise comparisons according to study variables are listed on Tables 1 and 2, respectively.

Accordingly, the distances between the canal’s entrance and alveolar crest, the canal’s entrance and mandibular basis, the canal’s outlet and alveolar crest, the canal’s outlet and mandibular basis, the canal’s outlet and lingual cortex, total bone height and total canal length were all significantly higher in males than females.

**Table 1:** Descriptives on all measurements

	Mean	Median	SD	Minimum	Maximum
Diameter Lingual Foramen	1.14	1.12	0.41	0.35	2.93
Distance Crest-Entrance	17.22	17.13	4.79	2.26	35.94
Distance Basis-Entrance	15.15	15.62	3.17	2.57	21.93
Distance Crest-Outlet	19.49	19.81	4.21	5.88	33.49
Distance Basis-Outlet	10.41	10.36	2.42	2.61	20.51
Distance Outlet-Buccal cortex	5.18	5.00	1.65	1.50	10.50
Distance Outlet-Lingual cortex	7.61	7.50	2.04	2.00	15.50
Total bone height	29.77	29.85	3.80	15.75	39.47
Total canal length	7.65	7.50	2.37	2.00	16.76

**Table 2:** Differences among genders according to study variables

	Test	Statistic	*p*
Diameter Lingual Foramen	Student’s t	-1.68	0.094
Distance Crest-Entrance	Student’s t	-4.40	< 0.001*
Distance Basis-Entrance	Welch’s t	-2.84ᵃ	0.005*
Distance Crest-Outlet	Student’s t	-5.70	< 0.001*
Distance Basis-Outlet	Student’s t	-2.56	0.011*
Distance Outlet-Buccal cortex	Student’s t	-0.68	0.497
Distance Outlet-Lingual cortex	Welch’s t	-3.83ᵃ	< 0.001*
Total bone height	Student’s t	-8.19	< 0.001*
Total canal length	Student’s t	-3.81	< 0.001*

Note. Hₐ µ_Female_ ≠ µ_Male_

Statistically significant at *p* < 0.05*

**Table 3:** Distribution and mean age of the study population according to dentition status

Dentiton Status	*n*	%	Mean Age
Fully Dentate	250	51,44%	33,04
Partial Dentate	174	35,80%	52,26
Edentulous	62	12,76%	61,55
Total	486		

The distribution of the study population according to dentition status and the corresponding mean ages are presented in Table 3, which demonstrates a progressive increase in mean age as the degree of edentulism advanced.

Regarding the number of lingual foramina, 62.8.% (*n* = 305) of scans revealed single foramen, 31.9% (*n* = 155) had double foramina, 4.9% (*n* = 24) displayed three foramina and only 0.4% of the study population (*n* = 2) had four foramina. The number of foramina among genders showed no statistically significant differences (Table 4).

**Table 4:** Number of foramina vs. Gender

Gender	Lingual foramina					
	One	Two	Three	Four	Total	Significance
Female	182	83	13	2	280	0.376
Male	123	72	11	0	206	
Total	305	155	24	2	486	

Similarly, number of foramina across different ages showed no statistically significant differences (*p* = 0.779). Spearman’s correlation analysis was employed to determine the relationship between patient age and two key morphometric measurements: diameter of the lingual foramen and total canal length. The results indicated no statistically significant correlations between age and the diameter of the lingual foramen. Conversely, the analysis revealed a statistically significant, yet very weak, negative association between patient age and total canal length (Table 5).

**Table 5:** Correlation Matrix

		Age
Total canal length	Spearman’s rho	-0.091
	Df	484
	*p*-value	0.045*
Diameter Lingual Foramen	Spearman’s rho	0.005
	Df	484
	*p*-value	0.911

Statistically significant at *p* < 0.05*

## Discussion

The present study provides a comprehensive CBCT-based morphometric evaluation of the mandibular lingual foramen and demonstrates substantial anatomical variability with clear clinical implications. The most prominent finding was the presence of statistically significant differences between male and female patients in nearly all vertical and horizontal bone measurements, including the distances between the lingual foramen, alveolar crest, and mandibular base. These findings confirm a distinct sexual dimorphism in mandibular anatomy, which is consistent with previously published data [5, 8, 9].

Similar results have been reported by Verma et al. and Moshfeghi et al., who observed significantly greater bone dimensions in male patients [8, 9]. These differences have been attributed to greater skeletal mass and more pronounced muscle attachments in males. Alqutaibi et al. further emphasized that males generally present with increased vertical bone height in the interforaminal region, potentially providing a wider surgical safety margin [5]. The consistency between our findings and those reported in both Turkish and/or international populations suggests that this anatomical dimorphism represents a stable biological characteristic rather than a population-specific phenomenon.

From a clinical perspective, this finding is particularly important. Previous studies have demonstrated that a reduced distance between the alveolar crest and the lingual foramen increases the risk of intraoperative complications, especially hemorrhage. Thambi and Aswath, as well as Şekerci et al., reported that female patients tend to present with narrower safety margins, predisposing them to a higher risk during implant placement or surgical manipulation in the anterior mandible [10, 11]. The present study supports these observations and reinforces the importance of considering gender as a critical factor during preoperative risk assessment.

Regarding the number of lingual foramina, the present study demonstrated that although a single foramen was the most common configuration, a considerable proportion of individuals exhibited multiple foramina. The detection of double, triple, and even quadruple foramina highlights the complex anatomy of the interforaminal region. These findings are consistent with those reported by Yildirim et al., who noted that multiple foramina are a frequent anatomical finding rather than an exception [12]. Variations in prevalence among studies, such as those reported by Trost et al., may be attributed to ethnic differences, sample size, or methodological variations in CBCT evaluation [13].

The high prevalence of multiple foramina observed in the present study further supports the conclusions of Alqutaibi et al. and Taschieri et al., who emphasized that accessory canals are common and clinically relevant [5, 14]. Similarly, the findings of Silvestri et al. and Mostafavi et al. demonstrated that lateral and accessory lingual canals represent consistent anatomical features rather than incidental findings [1, 15]. These anatomical variations may significantly increase the risk of unexpected bleeding if not properly identified preoperatively.

In terms of morphometric parameters, the mean foramen diameter observed in this study (1.14 ± 0.40 mm) is consistent with values reported by Gilis et al. and Katakami et al. [16, 17]. These findings indicate that the lingual foramen frequently accommodates vascular structures of sufficient caliber to cause clinically significant hemorrhage, underscoring the importance of detailed preoperative imaging.

The vertical position of the lingual foramen also showed considerable variation. In the present study, the mean distance from the foramen to the mandibular base was 15.15 mm, which is higher than values reported by Moshfeghi et al., Aoun et al., and Babiuc et al. [9, 18, 19]. This discrepancy may reflect population-specific anatomical characteristics or methodological differences. Nevertheless, the relatively superior location of the foramen suggests that the neurovascular bundle may be encountered at higher levels than anticipated, particularly during procedures such as bone graft harvesting or genioplasty.

The distance between the alveolar crest and the foramen, which averaged 17.21 mm, potentially indicates a safe zone for implant placement in the study population. However, this safety margin could be influenced by alveolar bone resorption. In the present study, while no significant correlation was observed between age and foramen diameter, a statistically significant but very weak negative correlation was found between age and canal length (*r* = -0.091). This finding should be interpreted with caution; while it may suggest an apparent migration of the foramen toward the alveolar crest as age increases, such a weak correlation implies that age alone is not a primary predictor of bone resorption. Instead, as discussed in the limitations, bone height changes are more likely associated with the individual’s dentition status and the duration of edentulism rather than chronological age.

Our results appear to align with those reported by Yu et al. and Padhye et al., who suggested that bone loss might increase the relative proximity of the lingual foramen to the alveolar crest [4, 7, 12]. However, given the marginal significance and weak correlation observed in our sample, further studies specifically controlling for dentition status are required to confirm if aging independently elevates surgical risk. Nevertheless, the persistence of vessel diameter regardless of bone height highlights the need for individualized preoperative planning, especially in patients with significant alveolar resorption.

Systematic reviews further support the clinical relevance of these findings. Rajkovic Pavlovic et al. emphasized that the anatomical variability of the lingual foramen precludes the establishment of universal safety margins and strongly advocated the use of CBCT for preoperative evaluation [3]. The present study corroborates these conclusions by demonstrating substantial interindividual variability in foramen number, size, and location.

Barbosa et al. also highlighted the importance of vessel diameter and anastomotic patterns in determining hemorrhagic risk during mandibular surgery [2]. In agreement with their findings, the present study demonstrated that foramen diameter remains unchanged with age, indicating that vascular risk persists even in elderly patients. When combined with age-related bone loss, this anatomical characteristic may significantly increase the likelihood of intraoperative complications.

Furthermore, the clinical implications of mandibular lingual foramen (MLF) anatomy extend beyond implant dentistry, as highlighted by a comprehensive systematic review and meta-analysis by Bernardi et al. [20]. These authors emphasized that the MLF is an almost universal anatomical feature containing vital neurovascular bundles that are at significant risk during various surgical procedures in the anterior mandible. Specifically, in procedures such as genioplasty, where horizontal osteotomies are performed, or in the harvesting of autogenous bone blocks from the symphyseal region, the risk of severe sublingual hemorrhage or neurosensory deficits is high if these foramina are not properly identified. Additionally, in the management of mandibular midline fractures, understanding the vertical distribution of the MLF is crucial for safe screw placement and internal fixation to avoid vascular trauma. Our morphometric findings, which provide detailed safety margins for different genders and ages, corroborate the necessity of preoperative CBCT evaluation advocated by Bernardi et al. to ensure patient safety in all maxillofacial interventions involving the interforaminal region.

Several limitations of this study should be acknowledged. First, the retrospective and cross-sectional nature of the study precludes the establishment of a longitudinal relationship between aging and bone resorption. While the relatively large sample size strengthens the reliability of the findings, the single-center design may limit the generalizability of the results to different ethnic populations. The exclusive use of CBCT imaging does not allow histological confirmation of canal contents, and very small accessory canals may remain undetected due to resolution limits. Furthermore, the study sample included fully edentulous, partially dentate, and completely dentate patients without stratification by dentition status. As edentulism is a well-established contributor to alveolar bone resorption and may independently influence measurements such as canal length and crest-to-foramen distances, the absence of this stratification represents a methodological limitation. It is essential to acknowledge that bone resorption is often more directly associated with tooth loss than chronological age alone. Future studies are encouraged to control for dentition status to isolate its specific effect on lingual foramen morphometry. Nevertheless, the high inter-observer reliability and standardized measurement protocol support the validity of the present results.

## Conclusion

The present study demonstrates that the mandibular lingual foramen exhibits significant anatomical variability, characterized by distinct sexual dimorphism and a high prevalence of multiple accessory canals. Female and elderly patients generally present with decreased vertical bone dimensions and narrower safety margins, which increases the potential risk of neurovascular injury during surgical interventions in the anterior mandible. Notably, vascular diameter remains consistent despite age-related bone resorption, suggesting that hemorrhagic risk persists in older populations. Therefore, routine preoperative evaluation using CBCT is strongly recommended to identify these specific anatomical variations and prevent intraoperative complications.

## Data Availability

The datasets used and/or analysed during the current study are available from the corresponding author on reasonable request.

## References

[1] Silvestri F, Nguyen JF, Hüe O, Mense C. Lingual foramina of the anterior mandible in edentulous patients: CBCT analysis and surgical risk assessment. Ann Anat. 2022;244:151982. 35882296 10.1016/j.aanat.2022.151982

[2] Barbosa DAF, de Mendonça DS, de Carvalho FSR, Kurita LM, de Barros Silva PG, Neves FS. Systematic review and meta-analysis of lingual foramina anatomy and surgical-related aspects on cone-beam computed tomography: a PROSPERO-registered study. Oral Radiol. 2022;38(1):1–16. 33609258 10.1007/s11282-021-00516-8

[3] Rajkovic Pavlovic Z, Stepovic M, Bubalo M, Zivanovic Macuzic I, Vulovic M, Folic N, et al. Anatomic variations important for dental implantation in the mandible—A systematic review. Diagnostics. 2025;15(2):155. 39857039 10.3390/diagnostics15020155 PMC11763380

[4] Padhye NM, Shirsekar VU, Rakhangi RS, Chalakuzhy PM, Joshi AV. Three-dimensional assessment of the mandibular lingual foramina with implications for surgical and implant therapy: A multicentre cross-sectional study. J Oral Biol Craniofac Res. 2023;13(2):186–90. 36688146 10.1016/j.jobcr.2023.01.002 PMC9852479

[5] Alqutaibi AY, Alassaf MS, Elsayed SA, Alharbi AS, Habeeb AT, Alqurashi MA, et al. Morphometric analysis of the midline mandibular lingual canal and mandibular lingual foramina: a cone beam computed tomography (CBCT) evaluation. Int J Environ Res Public Health. 2022;19(24):16910. 36554790 10.3390/ijerph192416910 PMC9779324

[6] Chen CM, Lee HN, Chen YT, Hsu KJ. Locating the mandibular lingula using cone-beam computed tomography: A literature review. J Clin Med. 2023;12(3):881. 36769529 10.3390/jcm12030881 PMC9917514

[7] Yu SK, Lim J, Bae CJ, Seo YS, Kim HJ. Morphometric analysis of the mandibular lingual foramina using cone-beam computed tomography in elderly Korean. Int J Morphol. 2022;40(3):688–96.

[8] Verma S, Krishna KS, Srishti, Shalini K, Kumari S, Sinha G. CBCT evaluation of lingual foramen and its anatomic variations in Northeast Indian population. J Pharm Bioallied Sci. 2023;15:S698–701. 37654303 10.4103/jpbs.jpbs_7_23 PMC10466637

[9] Moshfeghi M, Gandomi S, Mansouri H, Yadshoghi N. Lingual foramen of the mandible on cone-beam computed tomography scans: A study of anatomical variations in an Iranian population. Front Dent. 2021;18:20. 35965728 10.18502/fid.v18i20.6327 PMC9355889

[10] DevaThambi TJ, Aswath N. Evaluation of lingual foramen in the South Indian population using cone-beam computed tomography: A prospective cross-sectional study. J Pharm Bioallied Sci. 2024;16:S1140–46. 38882797 10.4103/jpbs.jpbs_21_24 PMC11174210

[11] Sekerci AE, Sisman Y, Payveren MA. Evaluation of location and dimensions of mandibular lingual foramina using cone-beam computed tomography. Surg Radiol Anat. 2014;36(9):857–64. 24831961 10.1007/s00276-014-1311-9

[12] Yildirim YD, Güncü GN, Galindo-Moreno P, Velasco-Torres M, Juodzbalys G, Kubilius M, et al. Evaluation of mandibular lingual foramina related to dental implant treatment with computerized tomography: a multicenter clinical study. Implant Dent. 2014;23(1):57–63. 24394340 10.1097/ID.0000000000000012

[13] Trost M, Mundt T, Biffar R, Heinemann F. The lingual foramina, a potential risk in oral surgery. A retrospective analysis of location and anatomic variability. Ann Anat. 2020;231:151515. 32229242 10.1016/j.aanat.2020.151515

[14] Taschieri S, Corbella S, Silnovic A, et al. Frequency and anatomic variability of the mandibular lingual foramina: a cone-beam CT study. BMC Med Imaging. 2022;22:12. 35057756 10.1186/s12880-022-00736-2 PMC8781116

[15] Mostafavi M, Zarch SHH, Eshghpour M, et al. Prevalence of accessory mental foramen and lateral lingual foramen using cone beam computed tomography: A single-center cross-sectional study. Oral Maxillofac Surg. 2024;28:1623–33. 39237742 10.1007/s10006-024-01289-0

[16] Gilis S, Dhaene B, Dequanter D, Loeb I. Mandibular incisive canal and lingual foramina characterization by cone-beam computed tomography. Morphologie. 2019;103(341):48–53. 30642812 10.1016/j.morpho.2018.12.005

[17] Katakami K, Mishima A, Kuribayashi A, Shimoda S, Hamada Y, Kobayashi K. Anatomical characteristics of the mandibular lingual foramina observed on limited cone-beam CT images. Clin Oral Implants Res. 2009;20(4):386–90. 19298292 10.1111/j.1600-0501.2008.01632.x

[18] Aoun G, Nasseh I, Sokhn S, Rifai M. Lingual foramina and canals of the mandible: anatomic variations in a Lebanese population. J Clin Imaging Sci. 2017;7:16. 28589055 10.4103/jcis.JCIS_15_17 PMC5433650

[19] Babiuc I, Tarlungeanu I, Pauna M. Cone beam computed tomography observations of the lingual foramina and their bony canals in the median region of the mandible. Rom J Morphol Embryol. 2011;52(3):827–82. 21892525

[20] Bernardi S, Bianchi S, Continenza MA, Macchiarelli G. Frequency and anatomical features of the mandibular lingual foramina: systematic review and meta-analysis. Surg Radiol Anat. 2017;39(12):1349–57. 28616681 10.1007/s00276-017-1888-x

